# Interactions between vaginal local cytokine IL-2 and high-risk human papillomavirus infection with cervical intraepithelial neoplasia in a Chinese population-based study

**DOI:** 10.3389/fcimb.2023.1109741

**Published:** 2023-05-15

**Authors:** Ruoxi Zhu, Wenhao Wang, Aiming Yang, Weihong Zhao, Wei Wang, Zhilian Wang, Jintao Wang, Yongli Hou, Xiaoqiang Su, Lili Zhang, Bo Feng, Jing Yang, Zhe Wang, Xiaofen Niu, Weiguo Lv, Zhican Qu, Min Hao

**Affiliations:** ^1^ Departments of Obstetrics and Gynecology, Second Hospital of Shanxi Medical University, Taiyuan, Shanxi, China; ^2^ Department of Medicine and Therapeutics, The Chinese University of Hong Kong, Prince of Wales Hospital, Hong Kong, Hong Kong SAR, China; ^3^ Hong Kong Institute of Diabetes and Obesity, The Chinese University of Hong Kong, Hong Kong, Hong Kong SAR, China; ^4^ Department of Epidemiology, School of Public Health, Shanxi Medical University, Taiyuan, Shanxi, China; ^5^ Department of Gynecologic Oncology, Women’s Hospital, School of Medicine, Zhejiang University, Hangzhou, Zhejiang, China

**Keywords:** cervical intraepithelial neoplasia, cytokines, IL-2, high-risk human papillomavirus infection, cervical cancer

## Abstract

**Background:**

Although interleukin-2 (IL-2) has long been associated with cancer development, its roles in the development of cervical cancer remains unclear. Few studies examined the associations between IL-2 and high-risk human papillomavirus (HPV) with risk of cervical intraepithelial neoplasia (CIN).

**Objective:**

We aimed to assess the association of IL-2 and high-risk HPV infection with risk of CIN as well as their interactions on the risk of CIN.

**Design:**

We performed a cross-sectional analysis of screening data in 2285 women aged 19-65 years who participated in an ongoing community-based cohort of 40,000 women in Shanxi, China in 2014-2015. Both categorical and spline analyses were used to evaluation the association between IL-2 in the local vaginal fluids and prevalence of CIN. In addition, 1503 controls were followed up until January 31, 2019), the nested case-control study design was adopted to evaluate the association of vaginal lavage IL-2 levels and the risk of CIN progression.

**Results:**

After adjusting for potential confounders, IL-2 levels were statistically inversely associated with prevalence of CIN (the 1st versus 4th quartile IL-2 levels: the respective odds ratio [OR] and 95% confidence intervals [CI] was: = 1.75 [1.37, 2.23] for CIN, 1.32 [1.01, 1.73] for CIN I, and 3.53 [2.26, 5.52] for CIN II/III). Increased IL-2 levels were inversely associated with prevalence of CIN (P-overall<0.01, P-nonlinearity<0.01 for CIN; P-overall<0.01, P-nonlinearity = 0.01 for CIN I; P-overall <0.01, P-nonlinearity = 0.62 for CIN II/III). The highest prevalence of CIN was observed in women with high-risk HPV, who also had the lowest IL-2 levels (P-interaction < 0.01). Nested case-control study observed an inverse association between IL-2 levels and risk of CIN progression (OR=3.43, [1.17, 10.03]).

**Conclusions:**

IL-2 levels in the local vaginal fluids were inversely associated with the risk of CIN in Chinese women either with or without high-risk HPV infection.

## 1 Introduction

Globally, the incidence of cervical cancer has declined due to the development of cervical cancer screening (Siegel et al., 2022). However, there has been an increasing trend in the incidence of cervical intraepithelial neoplasia (CIN) (Burness et al., 2020; Chen et al., 2020; Zhang et al., 2020). CIN is a precancerous lesion that pathologically reflects the dynamic changes of epithelial hyperplasia in cervical cancer and studying its mechanism is crucial for the prevention of cervical cancer (Curty et al., 2019; Kyrgiou and Moscicki, 2022; Kyrgiou et al., 2022). Previous studies have established the causal role of high-risk human papilloma virus (HPV) infection in the pathogenesis of CIN and cervical cancer (Bosch and de Sanjosé, 2003). However, in most women, the virus and diseased cells can be eliminated through autoimmune reactions, and only a few persistent infections eventually progress into cervical cancer. The immune system plays an important regulatory role in the development of CIN and cervical cancer caused by high-risk HPV infections (Sasagawa et al., 2012), as the occurrence and development of malignant diseases are closely related to the immune system.

Cytokines are low-molecular-weight soluble proteins that act as a medium of the immune system. They are induced by immunogens or other factors that are stimulated by immune or other cells, and have various functions such as regulating the development and function of the body’s immune and non-immune systems. HPV infection in the body is mainly depend on the cellular immunity of CD4+ T helper cells (Th) and cytotoxic T cells (CTL) to eliminate the virus (Brito et al., 2021). After the activation, CD4+ T cells differentiate into Th1 and Th2 cells under the stimulation of local cytokines. These cells secrete interleukin (IL)-2, IL-12, and interferon-γ (IFN-γ) that are mainly involved in cellular immunity. These cytokines can infiltrate in the female reproductive tract and participate in the regulation of the vaginal microenvironmental immune network. Changes in the immune network are closely related to the outcome of cervical lesions (Xu, 2014). IL-2 is an important cellular immune factor with anti-tumor effects (Jiang et al., 2016; Mu et al., 2020). It has been approved for the treatment of metastatic renal cell carcinoma and melanoma (Daniilidis et al., 2016). Recent reports have also demonstrated that changes in IL-2 levels play an important role in the development and progression of cervical cancer (Pang et al., 2014; Valle-Mendiola et al., 2016; Zhao et al., 2016). However, the specific mechanism remains unclear. It is unclear whether IL-2 and high-risk HPV have synergistic effects in the development of CIN and cervical cancer. Therefore, exploring the associations of cytokine IL-2 levels and CIN risk, and synergy with the high-risk HPV infection is important in timely blocking CIN progression.

Vaginal microecology includes vaginal flora, endocrine regulation, local immunity, and anatomy. Local immunity may play an important role in maintaining microecological balance. As the cervix is located in the vagina, the vaginal micro-ecological imbalance can have a direct impact on the cervix. Local immunity may also have a more significant role in cervical lesions than systemic immunity. Moreover, as the cervix can be easily exposed; it can easily adjust local immunity and prevent disease progression. However, limited research has been conducted on the effects of local immunity on diseases; Therefore, measuring the cytokine IL-2 levels in the local vaginal fluids can be an effective way of assessing immune changes that accurately reflect the cervix’s environment. This approach may prove to be more sensitive and specific than the blood and other body fluids.

To better understand the potential risk factors associated with CIN and to develop effective strategies for preventing its progression to cervical cancer, we launched a large population-based cervical cancer screening program and a prospective cohort study (Shanxi CIN Cohort) in Shanxi, China in 2014-2015 (Yang et al., 2018). In the study, we comprehensively assessed the associations of IL-2 levels in the local vaginal fluids with prevalence of CIN and interactions between IL-2 levels and high-risk HPV infection on CIN risk.

## 2 Material and methods

### 2.1 Study population

This study was conducted based on the data obtained from the Shanxi CIN Cohort Study in 2014-2015. The rationale, design, and methods of the cohort have been detailed elsewhere(Yang et al., 2018; Wang et al., 2021; Wang et al., 2022). Briefly, free cervical cancer screening was conducted for eligible women permanently residing in two counties of Shanxi Province, China between 2014 and 2015. A total of 40,000 women aged 19–65 years were included. All participants completed a demographic characteristic-related questionnaire and a Pap test according to a liquid-based cytology (LBC). All participants with abnormal Pap test results were referred for colposcopy and histopathological examination. Amongst 2,769 women diagnosed with atypical squamous cells of undetermined significance (ASC-US), 2,691 women underwent colposcopy and histopathological examination, and 78 (10 cases of abnormal gland cells and 68 cases of rejection) were excluded. Among the 2,691 women with pathological results, 1,890 tested negative, 564 with CIN grade 1 (CIN I), 171 with CIN II, 47 with CIN III, and 19 with cervical cancer. In this study, 19 cases of cervical cancer were excluded. Among the 1,890 women with negative colposcopy and pathological results, 1,503 were included in the final analysis and 387 women were excluded because they had not fully completed the three parts of the medical examination, i.e., an in-person interview, physical examination, and clinical examination. The basic characteristics of the included and excluded women with negative pathological results are presented in **Supplementary Table 1**. Thus, a total of 2,285 women aged 49.2 ± 9.0 years (mean± standard deviation [SD]) was included in the present analysis.

Additionally, 1503 women of the control group had followed up until January 3, 2019. Twenty-five women (16 CIN I, 6 CIN II, 3 CIN III) with abnormal biopsy results were selected as the case group. The research carried out a follow-up on women with negative pathological examination results. During the follow-up period, patients who progressed to CIN I, CIN II or CIN III were the case group, and those who did not progress to the control group were matched at a ratio of 1:3. The matching condition included no disease progression occurred on the latest follow-up date of the case group, age ± 1 year old, and high-risk HPV infection. We finally matched to 25 cases and 75 controls. The study was approved by the ethical committees of the Second Hospital of Shanxi Medical University, and registered in the Chinese Clinical Trial Register (ChiCTR) (registration number: ChiCTR-ROC-15006479).

### 2.2 Data collection

#### 2.2.1 Demographic characteristics and collection of factors related to cervical lesions

Face-to-face interviews were conducted by trained interviewers using a standardized and structured questionnaire. The interviewers were trained to administer the questionnaire in a standardized style. The survey mainly includes the demographic characteristics, such as age, educational years, smoking, alcohol drinking, and yearly income; characteristics of cervical lesion-related factors, such as parity, first sexual intercourse age, and family history of cancer.

#### 2.2.2 Specimen collection

##### 2.2.2.1 Collection of cervical cell samples

All participants were instructed to abstain from sexual intercourse and not perform vaginal lavage or medications for 48 h prior to the sampling. After exposing the cervix, the cervical detachment cells were collected by rotating the cervical cell brush five times, and the sampler was rinsed 10 times with the preservation solution, and then tightened after capping. The samples were stored at 4°C and tested within 1 week. Dissatisfactory cervical cell specimens were re-acquired after reading the films, and satisfactory specimens were obtained from each participant.

##### 2.2.2.2 Operation procedure of HPV classification test specimen collection

The HPV test special brush was inserted into the cervical canal, and gently rotated for 3-5 turns; the brush head was left in the sample tube, and stored in −20°C refrigerator for inspection.

##### 2.2.2.3 Vaginal lavage fluid collection

The vaginal lavage fluid was taken before the colposcopy biopsy. The speculum was opened, 5 ml of normal saline was taken using a disposable sterile syringe, 1/3 of the vagina and cervix was rinsed, and 5 ml of vaginal lavage was extracted, stored in a sterile centrifuge tube, and tested for the cytokine IL-2 levels. The collected vaginal lavage fluid was centrifuged for 10 min (2000 rpm), and the supernatant was dispensed and stored inside a refrigerator at −80°C until use.

##### 2.2.2.4 Cervical tissue

Cervical tissue specimens were collected by a physician with colposcopy. The cervical mucus was gently wiped using a sterile cotton ball, and the suspicious part of cervix was taken using a colposcopy sterile biopsy forceps. The clamped tissue was immediately sent to the pathology department of our hospital for pathological diagnosis.

#### 2.2.3 Clinical laboratory tests

##### 2.2.3.1 Cervical Pap cytology specimen testing

All Pap tests were performed using an LBC method. Two cytopathologists from the Second Hospital of Shanxi Medical University performed the cytologic evaluation according to Bethesda System 2001 terminology. All slides with abnormal cytological results were further reviewed by a senior cytopathologist blinded on the previous pathological results for quality control.

##### 2.2.3.2 Colposcopy and cervical histological examination

Colposcopy was performed by gynecological specialists at the Second Hospital of Shanxi Medical University according to a standard protocol, within 12 weeks from the Pap test. Video colposcopy was performed using the SLC-2000 device (Shenzhen Goldway Company, Shenzhen, Guangdong, China). During the colposcopy, the cervix was divided into quadrants, and each quadrant was examined. All visually abnormal areas were biopsied, and the quadrants without a visible lesion were biopsied at the squamocolumnar junction (“random biopsy”). Women with abnormal cytological results and negative or inadequate colposcopic findings also underwent endocervical curettage. Cervical biopsy and endocervical curettage results were evaluated by two gynecologic pathologists from the Second Hospital of Shanxi Medical University. The cases were classified as negative, CIN I, CIN II, CIN III, or squamous cell carcinoma. The pathologists were blinded to the Pap test results when they read the cervical biopsy or endocervical curettage tissue specimens. If the two pathologists had different diagnoses, the cases were reviewed by a third senior pathologist. The three pathologists reviewed difficult or equivocal cases together to reach a consensus diagnosis.

##### 2.2.3.3 HPV typing test

HPV genotyping by HybriMax was performed using an HPV GenoArray Test Kit (HybriBio Ltd) with the residual Pap test specimens. HPV types were divided into high-risk HPV types and others (including low-risk HPV types and negative) based on their oncogenic potential. This assay can identify 21 types of HPV, including 15 high-risk (16, 18, 31, 33, 35, 39, 45, 51, 52, 53, 56, 58, 59, 66, and 68) and 6 low-risk (6, 11, 42, 43, 44, and CP8304) types through the use of a flow-through hybridization technique performed with a TC-96/G/H6 HPV DNA Amplification Analyzer and an HMM-2 fast nucleic acid molecule hybridization instrument (HybriBio Ltd).

##### 2.2.3.4 IL-2 test

IL-2 levels were measured using the enzyme-linked immunosorbent assay (ELISA) kits (Jinma company in Shanghai) according to the manufacturer’s instructions.

### 2.3 Statistical analysis

The EpiData 3.1 software was used to enter and manage the datasets. Descriptive statistics were used to describe the frequency, proportion, mean, median, and quartile and the characteristics of demographic and cervical lesion-related factors. Participants’ characteristics were examined for significant difference(s) using the Pearson’s chi-square test for categorical variables and t-test for continuous variables. Logistic regression models were used to test odds ratios (ORs) and confidence intervals (CIs) of CIN II/III for each IL-2 quartile compared with the highest quartile. Tests of linear trends across the increasing quartiles of IL-2 levels were conducted by assigning the medians of quartiles treated as continuous variables. Interaction between IL-2 levels and high-risk HPV infections with prevalence of CIN were evaluated by including the terms in the model. After the crude analysis, we adjusted for the demographic characteristics such as age, educational level, yearly income, smoking, alcohol drinking, parity, first sexual intercourse age, family history of cancer, and high-risk HPV infection fitted simultaneously. Smoking was defined as those who smoked at least 1 cigarette/day in the past 6 months. Alcohol drinking was defined as those who drank hard liquor, beer, or wine at least 1/week in the past 6 months.

We further performed the spline analysis with a three-knot (25th, 50th, and 75th percentiles) restricted cubic spline function(Desquilbet and Mariotti, 2010; Yang et al., 2018) to determine the dose-response association between log-transformed IL-2 levels and prevalence of CIN. All statistical analyses were performed with SAS software version 9.3 (SAS Institute Inc.). All reported P-values were derived based on the two-sided tests with a significance level of 0.05.

## 3 Results

**Table 1** shows the characteristics of 2,285 women who underwent a cervical histologic examination. The proportion of women with CIN accounted for 34.2% of the analyzed participants including 564 with CIN I, 171 with CIN II, and 47 with CIN III, The respective proportion in women with and without CIN were 64.3% and 68.7% age above 45 years, 23.1% and 19.2% for yearly income above 30,000 yuan, 2.4% and 4.4% for alcohol drinking, and 13.3% and 8.8% for family history of cancer. Women with CIN were more likely infected with high-risk HPV versus those without CIN (39.5% versus 28.5%). Amongst 782 women with CIN, 72.1% were CIN I with high-risk HPV (n=564) with high-risk HPV prevalence of 29.6%, only 27.9% of women were in CIN II+ (n=218) with the prevalence of high-risk HPV of 65.1%. The median IL-2 level in the vaginal lavage fluid among women with and without CIN was 171.00 (132.93–221.22) pg/ml and 186.00 (151.00–226.38) pg/ml, respectively.

**Table 1:** Characteristics of 2,285 women with cervical histologic examination^1^ (%).

Characteristics	CIN		Total	*P value* ^2^
	No	Yes		
**No. of participants**	1503 (65.8)	782 (34.2)	2285 (100.0)	
**Age, years**	49.4 ± 8.9	48.6 ± 9.2	49.2 ± 9.0	
≤ 45	470 (31.3)	279 (35.7)	211 (9.2)	0.03
>45	1033 (68.7)	503 (64.3)	636 (27.9)	
Education, years				
≤9	1259 (86.2)	675 (86.3)	1970 (86.2)	0.16
>9	208 (13.8)	107 (13.7)	315 (13.8)	
Yearly income, ¥				
<30000	1214 (80.8)	601 (76.9)	1815 (79.4)	0.03
≥30000	289 (19.2)	181 (23.1)	470 (20.6)	
Smoking				
No	1474 (98.1)	763 (97.6)	2237 (97.9)	0.43
Yes	29 (1.9)	19 (2.4)	48 (2.1)	
Alcohol drinking				
No	1437 (95.6)	763 (97.6)	2200 (96.3)	0.02
Yes	66 (4.4)	19 (2.4)	85 (3.7)	
Parity				
<3	1110 (79.3)	578 (73.9)	1688 (73.9)	0.98
≥3	393 (26.1)	204 (26.1)	597 (26.1)	
First sexual intercourse age, years				
<23	847 (56.4)	464 (59.3)	1311 (57.4)	0.17
≥23	656 (43.6)	318 (40.7)	974 (42.6)	
Family history of cancer				
No	1370 (91.2)	678 (86.7)	2048 (89.6)	0.01
Yes	133 (8.8)	104 (13.3)	237 (10.4)	
High-risk HPV infection				
Negative	1075 (71.5)	473 (60.5)	1548 (67.7)	<0.01
Positive	428 (28.5)	309 (39.5)	737 (32.3)	
**IL-2 (pg/ml)**	186.00 (151.00-226.38)	171.00 (132.93-221.22)	182.00 (144.26-224.90)	<0.01

^1^Values are mean ± SD for normally distributed variables, median (25–75 percentiles) for skewed variables, or n (%) for categoric variables. CIN, cervical intraepithelial neoplasia

^2^P values for differences between groups were obtained from the chi-square test for categoric variables, and t-test for continuous variables.

**Table 2** presents the associations of IL-2 levels and high-risk HPV infection with prevalence of CIN in 2,285 women. After fully adjusting the demographic, lifestyle, and other covariates including high-risk HPV infection, IL-2 levels were inversely associated with prevalence of CIN. The 1st quartile levels of IL-2 had a 75% increased odds of CIN than the 4th quartile (OR=1.75, 95% CI: 1.37, 2.23, P-trend < 0.01). Compared with women without high-risk HPV infections, those with high-risk HPV infection had an increased odds of CIN (OR = 1.58, 95% CI: 1.31, 1.90). In stratified analysis by CIN subgroups, a statistically significant association between IL-2 levels and prevalence of CIN I was observed (1st versus 4th quartile: OR = 1.32; 95% CI: 1.01 1.73) while an inverse association was observed for CIN II/III (1st versus 4th quartile: OR = 3.53; 95% CI: 2.26, 5.52).

**Table 2:** The associations between IL-2 levels and high-risk HPV infection with prevalence of cervical intraepithelial neoplasia (CIN)among 2,285 women^1^.

	Odds Ratios (95% CIs)^2^		
	Model 1	Model 2	Model 3
CIN			
IL-2 (pg/ml)			
Q1 (≤ 144.26)	1.72 (1.35-2.19)	1.78 (1.39-2.26)	1.75 (1.37-2.23)
Q2 (144.27-182.00)	0.96 (0.75-1.24)	1.02 (0.79-1.31)	1.00 (0.77-1.28)
Q3 (182.01-224.90)	0.77 (0.59-0.99)	0.79 (0.61-1.02)	0.79 (0.61-1.02)
Q4 (≥ 224.91)	1.00 (Reference)	1.00 (Reference)	1.00 (Reference)
*P- trend*	< 0.01	< 0.01	< 0.01
High-risk HPV infection			
Positive	1.64 (1.37-1.97)	1.61 (1.33-1.93)	1.58 (1.31-1.90)
Negative	1.00 (reference)	1.00 (reference)	1.00 (reference)
CIN I			
IL-2 (pg/ml)			
Q1 (≤ 144.26)	1.32 (1.01-1.72)	1.33 (1.02-1.73)	1.32 (1.01-1.73)
Q2 (144.27-182.00)	0.85 (0.65-1.12)	0.86 (0.66-1.14)	0.86 (0.66-1.14)
Q3 (182.01-224.90)	0.67 (0.51-0.89)	0.68 (0.51-0.90)	0.68 (0.51-0.90)
Q4 (≥ 224.91)	1.00 (Reference)	1.00 (Reference)	1.00 (Reference)
*P- trend*	0.08	0.07	0.07
High-risk HPV infection			
Positive	1.06 (0.86-1.31)	1.05 (0.85-1.31)	1.04 (0.83-1.29)
Negitive	1.00 (Reference)	1.00 (Reference)	1.00 (Reference)
CIN II/III			
IL-2 (pg/ml)			
Q1 (≤ 144.26)	3.64 (2.37-5.58)	3.69 (2.40-5.70)	3.53 (2.26-5.52)
Q2 (144.27-182.00)	1.51 (0.95-2.41)	1.59 (0.99-2.56)	1.48 (0.91-2.41)
Q3 (182.01-224.90)	1.22 (0.76-1.98)	1.28 (0.78-2.07)	1.27 (0.77-2.09)
Q4 (≥ 224.91)	1.00 (Reference)	1.00 (Reference)	1.00 (Reference)
*P- trend*	< 0.01	< 0.01	< 0.01
High-risk HPV infection			
Positive	4.69 (3.48-6.31)	4.31 (3.18-5.86)	4.39 (3.20-6.01)
Negative	1.00 (Reference)	1.00 (Reference)	1.00 (Reference)

^1^Values are ORs (95% CIs) obtained from logistic regression analysis, using the highest intake group as the reference, unless otherwise indicated. CIN, cervical intraepithelial neoplasia.

^2^Model 1: unadjusted.

Model 2: adjusted for age, educational level, yearly income, smoking, alcohol drinking, parity, first sexual intercourse age, and family history of cancer

Model 3: adjusted for Model 2 + high-risk HPV infection

**Figure 1:**
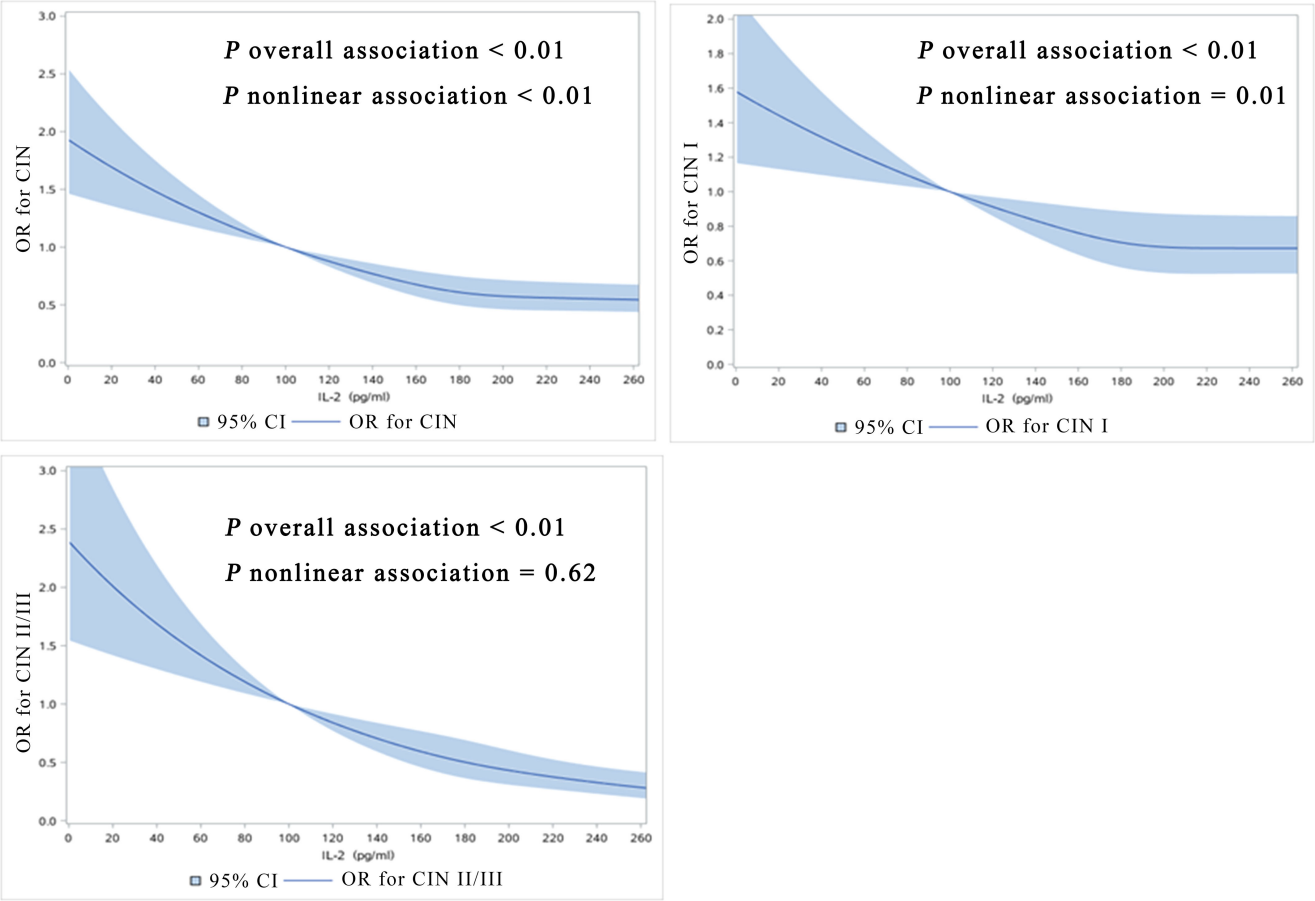
Dose-response relationship between IL-2 levels with prevalence of CIN, CIN I, CIN II/III (restricted cubic spline models). The solid line represents the OR from the adjusted restricted cubic polynomial spline. The shaded area is the 95% CI. Adjusted for age, educational level, yearly income, smoking, alcohol drinking, parity, first sexual intercourse age, family history of cancer, and high-risk HPV infection. CIN, cervical intraepithelial neoplasia.

Among the 2285 women, 1563 were high-risk HPV negative, and 15 of them were low-risk HPV infected. The mean ± SD of IL-2 in 15 women with low-risk HPV infection was 181.64 ± 49.46pg/ml with the median was 178.55pg/ml. Among 722 women with high-risk HPV infections the mean ± SD of 194.58 ± 99.86pg/ml; 1548 negative cases, with a mean ± SD of 204.82 ± 108.21pg/ml and a median of 183.60 pg/ml. We did not observe he statistically significant difference between negative, low-risk HPV and high-risk HPV patients (P = 0.075). After adjusting for all potential confounders including high-risk HPV infections, we observed the inverse linear dose-response associations between IL-2 levels and prevalence of CIN (P overall <0.01, P nonlinear < 0.01, CIN I), CIN I (P overall <0.01, P nonlinear= 0.01), and CIN II/III (P overall <0.01, P nonlinear = 0.62) (**Figure 1**).

**Figure 2:**
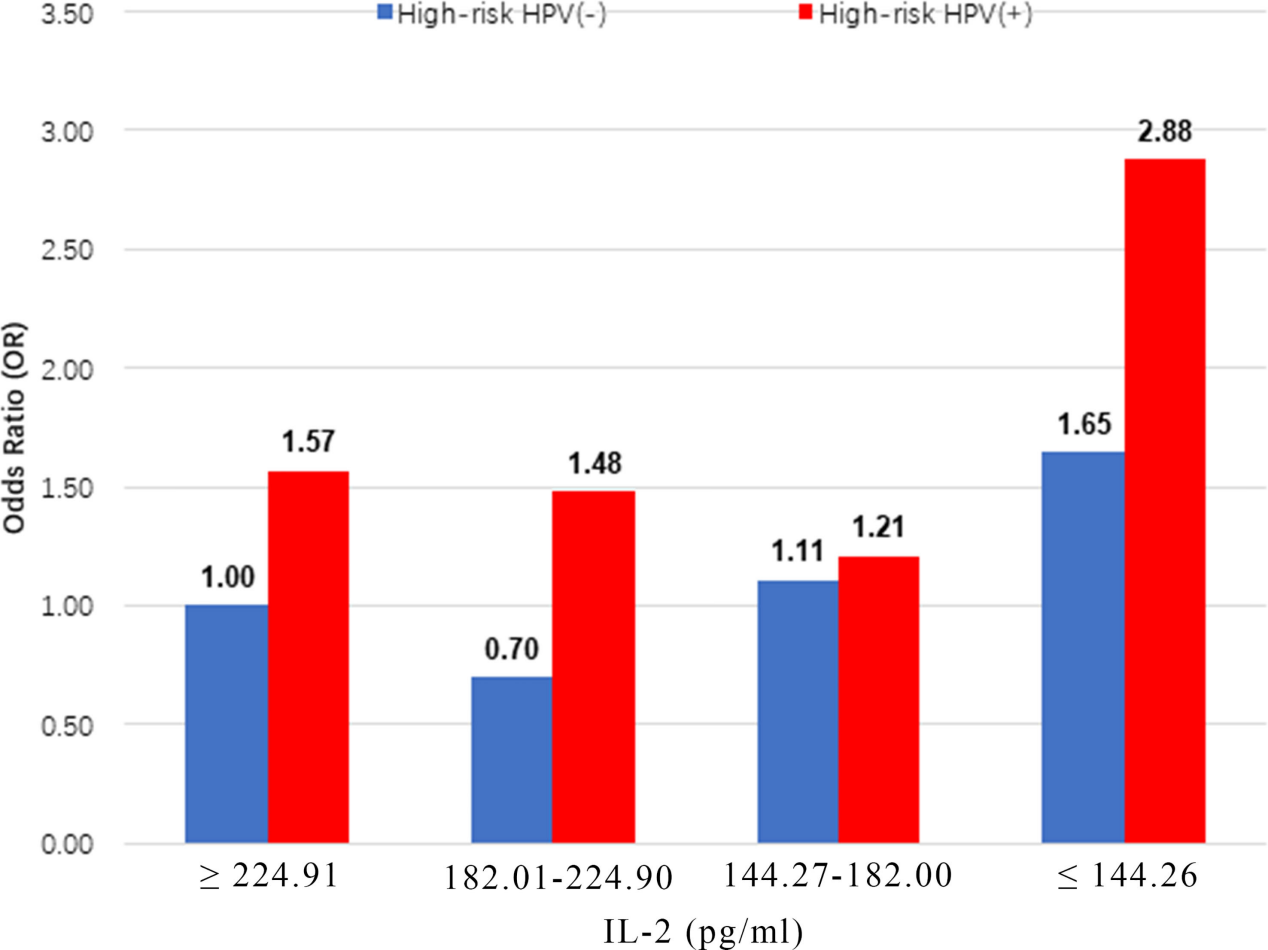
Multiplicative interaction between IL-2 level and high-risk HPV infection with prevalence of CIN. ORs are adjusted by age, educational level, yearly income, smoking, alcohol drinking, parity, first sexual intercourse age, family history of cancer.IL-2 ORs for CIN: 1.00 (reference), 0.70 (95% CI: 0.51, 0.96), 1.11 (95% CI: 0.81, 1.51), 1.65 (95% CI: 1.22, 2.22), 1.57 (95% CI: 1.08, 2.29), 1.48 (95% CI: 1.01, 2.17), 1.21 (95% CI: 0.83, 1.76) and 2.88 (95% CI: 2.02,4.11). CIN, cervical intraepithelial neoplasia.

The highest prevalence of CIN was observed in women with high-risk HPV infections, who also had the lowest IL-2 levels (≤144.26 pg/ml; OR = 2.88, 95% CI: 2.02, 4.11) (**Figure 2**). IL-2 was associated with a lower prevalence of high-risk HPV infection in Chinese women of this cohort (OR = 0.99; 95% CI: 0.99, 1.00; P=0.03) (**Supplementary Table 2**).

In the nested case-control study, we compared IL-2 expression levels before and after follow-up. The mean ± SD of IL-2 level before follow-up in the progression group (n=25) was 205.4 ± 93.84 pg/ml, the median value was 202.0 pg/ml. The mean ± SD IL-2 level at end of follow-up was 142.0 ± 58.44 pg/ml (media= 137.0 pg/ml). The mean ± SD of IL-2 level before follow-up in the non-progressive population (n=75) was 207.1 ± 123.8 pg/ml (median = 177.0 pg/ml). The mean ± SD of IL-2 level at end of follow-up was 170.3 ± 42.11 pg/ml (median=172.0 pg/ml). After adjusting the potential confounders, those with local vaginal IL-2 ≤ 169.00pg/ml (median) had a 3.43 (1.17-10.03) times risk of progression to CIN than those with IL-2>169.00pg/ml (**Table 3**). We also observed that IL-2 was inversely associated with risk of high-risk HPV infection (OR = 0.20; 95% CI: 0.09, 0.47) (**Supplementary Table 3**).

**Table 3:** Logistic regression analysis of IL-2 and risk of CIN in follow-up (n=100).

	ORs (95% CIs)^1^
IL-2 (pg/ml)	
≤ 169.00 (median)	3.43 (1.17-10.03)
>169.00 (median)	1.00 (Reference)

^1^adjusted for age, educational level, yearly income, smoking, alcohol drinking, parity, first sexual intercourse age, and family history of cancer.

## 4 Discussion

In this large-scale population-based study, we found that IL-2 levels in the local vaginal were associated with higher prevalence of CIN, with a non-linear dose-response associations in Chinese women in the Shanxi CIN cohort. Women with high-risk HPV infections and the lowest IL-2 levels in the vaginal lavage fluid had the highest prevalence of CIN. These results indicate the potential role of IL-2 levels in vaginal lavage in the development of CIN, regardless of high-risk HPV infections.

Previous studies on CIN have mostly focused on systemic immunity, however, studies on the cervical microenvironment have been limited. The mucosal immunity of the genitals is the first line of defense in the female genital tract. It has cellular and humoral immunity that is regulated by pathogens and cytokines. As an important component of the body’s immunity, the expression of local cytokines can better reflect the local immune status of the genital tract (Mitra et al., 2021). The incidence of CIN and cervical cancer is associated with high-risk HPV infection, and local immune capacity and systemic immunity may change after high-risk HPV infection (Arany et al., 2002; Torres-Poveda et al., 2016). cytokines have a high affinity for cell surface receptors, serving as an important structural basis for their biological function. *Passmore J A et al.* (Passmore et al., 2006) showed that local cytokines are more representative of local immune status than serum cytokines. Furthermore, vaginal lavage fluid collection is noninvasive, and specimens are readily available.

4.1 Association between IL-2 level and CIN risk

The role of IL-2 in the development of CIN and cervical cancer remains unclear (Meng and Song, 2019; Chen et al., 2021). Evidence from previous studies on the association of increased IL-2 level with decreased risk of CIN were certainly consistent. IL-2 can induce the differentiation of natural killer cells (NK) and CTL and exert anti-tumor effects (Banchereau et al., 2012). The activity of NK and CTL is decreased in the absence of IL-2, promoting the development of tumors including cervical cancer (Lieberman and Tsokos, 2010; Valle-Mendiola et al., 2016; Abel et al., 2018). Studies have shown that IL-2 inhibits the proliferation of cervical cancer cells at high doses (Valle-Mendiola et al., 2014). A recent study showed that serum IL-2 levels were significantly reduced in high-grade CIN compared with low-grade CIN (Daniilidis et al., 2016). Our study using baseline data also showed that even after adjusting for high-risk HPV and other potential confounders, vaginal local IL-2 was associated with the odds of CIN (OR=1.75 [1.37-2.23]). In addition, women with lower vaginal IL-2 levels were also more likely to develop into CIN in our follow-up data.

IL-2 has been widely used as a treatment for several other tumors by promoting CTL proliferation and activity (Kang et al., 2012; Abel et al., 2018; Dutcher and Wiernik, 2018; Ngai et al., 2018; Rolley et al., 2018). Thus, the specific role of IL-2 is also being explored in cervical cancer. However, whether low-level IL-2 promotes the occurrence of CIN remains unclear. Furthermore, the definition of the low level of IL-2 is not well-defined. More research is needed to confirm these results.

4.2 Interactions between cytokine IL-2 levels and high-risk HPV infection

High-risk HPV infection is an essential factor in the development of cervical cancer (Yu et al., 2022). In this study, we observed a lower levels of IL-2 in vaginal lavage fluids and the interaction of high-risk HPV infection with the risk of CIN. The association between IL-2 levels and the development of CIN and the interaction between IL-2 levels and high-risk HPV infection has not been fully elucidated in epidemiological studies. The exact associations between IL-2 levels and high-risk HPV infection in the vaginal microenvironment with the development of cervical cancer remains unclear. Most HPV infections are transient, and 90% of HPV-DNA is negative in women with normal immune function after 2 years. Even in women with CIN, high-risk HPV infection has a higher natural outcome rate if followed up for a sufficient period of time. After the high-risk HPV infection, the virus is mainly eliminated by the body’s immune response, and IL-2 is the most prominent anti-infective (Barros et al., 2018). IL-2 has been shown to act as an adjuvant vaccine to enhance mucosal immunity and form long-term memory responses (Kim et al., 2013; Ma et al., 2015). Studies by *Bashaw et al.* (Bashaw et al., 2017) showed that IL-2 may play an anti-high-risk HPV role in the early stage of infection. When IL-2 level decreases, it may lead to the decline of host anti-tumor immunity, which is not conducive to clearing HPV infection, and then leads to disease progression. *Paradkar et al.* (Paradkar et al., 2014) reported that IL-2 levels were lower in the peripheral blood of women with high-risk HPV infection than those without.

The studies mentioned above, decreased IL-2 levels in the vaginal microenvironment may affect the clearance of high-risk HPV and lead to persistent infection of high-risk HPV, while persistent infection of high-risk HPV may cause the decreased secretion of IL-2 by local production of E6, E7, etc. (Trujillo-Cirilo et al., 2021), which promotes the development of cervical cancer. Such interactions may be one of the underlying mechanisms that promote the development of CIN and cervical cancer.

Several advantages and limitations should be considered when interpreting these results. First, this large-scale study ensures that statistically significant differences can be detected. Second, an objective assessment of IL-2 levels, high-risk HPV, and other cervical cancer-related clinical examinations such as Pap test colposcopy, and cervical biopsy was conducted. Pathological results regarded as the “golden standard” instead of general cytology were used in classifying the participants. Third, potential confounding factors were carefully and comprehensively measured and analyzed to minimize the possibility of bias. Fourth, the lower high-risk HPV positive rate for CIN patients in this study may be due to differences in the study population or variations in pathological diagnosis and laboratory examination. Lastly, the dose-response relationship between IL-2 levels and CIN as well as their interactions based on high-risk HPV infection were evaluated. The main limitation of this study is that the participants were regionally confined. Since the information in this study is based on the screening and investigation of group research in Shanxi Province, regional differences could not be overcome.

In summary, IL-2 levels in local vaginal fluid were independently associated with risk of CIN in this population-based study. Given the significant interactions between IL-2 and high-risk HPV infection with CIN risk, future large-scale prospective studies should focus on developing a strategy to determine these interactions.

## Data availability statement

The raw data supporting the conclusions of this article will be made available by the authors, without undue reservation.

## Ethics statement

This study was approved by the ethical committees of the Second Hospital of Shanxi Medical University, and registered in the Chinese Clinical Trial Register (ChiCTR) (registration number: ChiCTR-ROC-15006479). The patients/participants provided their written informed consent to participate in this study.

## Author contributions

RZ and WHW designed the analysis pipeline and wrote the manuscript. AY revised the manuscript critically. WZ, WW, ZLW, and JW performed atherosclerotic plaque collection and sorting. BF, JY, ZW, and XN created figures. MH reviewed and revised the paper in detail. All authors contributed to the article and approved the submitted version.
