# Supplementary material for: Interactions between vaginal local cytokine IL-2 and high-risk human papillomavirus infection with cervical intraepithelial neoplasia in a Chinese population-based study
Source: Front Cell Infect Microbiol. 2023 May 15;13:1109741. doi: 10.3389/fcimb.2023.1109741 (PMC10225571; doi:10.3389/fcimb.2023.1109741)
Supplement: Supplementary file 1 [file Table_1.docx]

Supplementary Material

# 1 Supplementary Table 1: The socio-demographic characteristics of included and exclude women with normal cervix

| **Characteristics** | **Women with normal cervix (n= 1890)** | | ***P value*** |
| --- | --- | --- | --- |
|  | **Included**  **(n= 1,503)** | **Excluded**  **(n= 387)** |  |
| **Age (years)** |  |  |  |
| < 35 | 114 (7.6) | 37 (9.6) | 0.14 |
| 35-44 | 317 (21.1) | 90 (23.3) |  |
| 45-54 | 571 (38.0) | 154 (39.8) |  |
| 55-64 | 495 (32.9) | 106 (27.4) |  |
| > 65 | 6 (0.4) | 0 (0.0) |  |
| **Education , years** |  |  |  |
| 0-6 | 306 (20.4) | 66 (17.1) | 0.25 |
| 7-9 | 633 (42.1) | 178 (46.0) |  |
| > 9 | 564 (37.5) | 143 (37.0) |  |
| **Yearly income, ¥** |  |  |  |
| <10000 | 173 (11.5) | 18 (4.7) | <0.01 |
| 10000-30000 | 738 (49.1) | 137 (35.4) |  |
| >30000 | 592 (39.4) | 232 (59.9) |  |
| **Smoking** |  |  |  |
| No | 1474 (98.1) | 378 (97.7) | 0.62 |
| Yes | 29 (1.9) | 9 (2.3) |  |
| **Marital status** |  |  |  |
| Married | 1408 (93.7) | 368 (95.1) | 0.30 |
| Others | 95 (6.3) | 19 (4.9) |  |

**2 Supplementary Table 2:** Logistic regression analysis of association IL-2 and HR-HPV in baseline population

|  | **ORs (95% CIs)** |
| --- | --- |
| **IL-2 (pg/ml)** | 0.99(0.99-1.00) |

**3 Supplementary Table 3:** Logistic regression analysis of risk of IL-2 and HR-HPV in follow-up population

|  | **ORs (95% CIs)** |
| --- | --- |
| **IL-2 (pg/ml)** |  |
| Q1 (≤169.00) | 1.00 (Reference) |
| Q2 ( >169.00) | 0.20 (0.09-0.47) |
